# Synthesis and Characterization of Hybrid Materials Derived from Conjugated Copolymers and Reduced Graphene Oxide

**DOI:** 10.3390/polym14235292

**Published:** 2022-12-03

**Authors:** Alexandros Ch. Lazanas, Athanasios Katsouras, Michael Spanos, Gkreti-Maria Manesi, Ioannis Moutsios, Dmitry V. Vashurkin, Dimitrios Moschovas, Christina Gioti, Michail A. Karakassides, Vasilis G. Gregoriou, Dimitri A. Ivanov, Christos L. Chochos, Apostolos Avgeropoulos

**Affiliations:** 1 Department of Materials Science Engineering, University of Ioannina, 45110 Ioannina, Greece; 2 National Hellenic Research Foundation (NHRF), 48 Vassileos Constantinou Avenue, 11635 Athens, Greece; 3 Institut de Sciences des Matériaux de Mulhouse—IS2M, CNRS UMR7361, 15 Jean Starcky, 68057 Mulhouse, France; 4 Faculty of Chemistry, Lomonosov Moscow State University, 9MSU, GSP-1, 1-3 Leninskiye Gory, 119991 Moscow, Russia; 5 Institute of Problems of Chemical Physics, Russian Academy of Sciences, Chernogolovka, 142432 Moscow, Russia

**Keywords:** hybrid materials, conjugated copolymers, rGO, HOMO, LUMO, indacenothiophene, indacenothienothiophene, cyclic voltammetry

## Abstract

In this study the preparation of hybrid materials based on reduced graphene oxide (rGO) and conjugated copolymers is reported. By tuning the number and arrangement of thiophenes in the main chain (indacenothiophene or indacenothienothiophene) and the nature of the polymer acceptor (difluoro benzothiadiazole or diketopyrrolopyrrole) semiconducting copolymers were synthesized through Stille aromatic coupling and characterized to determine their molecular characteristics. The graphene oxide was synthesized using the Staudenmaier method and was further modified to reduced graphene oxide prior to structural characterization. Various mixtures with different rGO quantities and conjugated copolymers were prepared to determine the optoelectronic, thermal and morphological properties. An increase in the maximum absorbance ranging from 3 to 6 nm for all hybrid materials irrespective of the rGO concentration, when compared to the pristine conjugated copolymers, was estimated through the UV-Vis spectroscopy indicating a differentiation on the optical properties. Through voltammetric experiments the oxidation and reduction potentials were determined and the calculated HOMO and LUMO levels revealed a decrease on the electrochemical energy gap for low rGO concentrations. The study indicates the potential of the hybrid materials consisting of graphene oxide and high band gap conjugated copolymers for applications related to organic solar cells.

## 1. Introduction

Over the last two decades graphene [1] has made an impressive impact on current technology due to its remarkable properties. The oxidized form of graphene, namely graphene oxide (GO), has been known for almost two centuries [2], and despite its impressive features, it lacks the electronic properties of graphene due to the mixed sp^3^ and sp^2^ orbital attributed to the oxygen groups. Many efforts have been conducted to reduce the oxygen groups using reductive substances such as NaBH_4_ etc. [3,4]. This allotropic form of carbon is generally known as reduced graphene oxide (rGO) and constitutes a fairly good compromise between the outstanding electronic properties of graphene and the excellent solubility of graphene oxide. This fact is allocated to the reduction process which is not exclusively completed (not 100% yield of the process) and the produced rGO still retains some of the aforementioned oxygen groups that enable stable dispersions with hydrophobic interactions, since rGO is not water-soluble similar to its precursor [5].

The above-mentioned characteristics strongly suggest that rGO could readily form films for a great number of applications such as organic photovoltaics [6,7], sensors [8] etc. To that end, many groups have endeavored the combination of conjugated polymers and rGO [9,10,11] to address the fact that their possible π-π stacking with graphene and rGO enables the fine-tuning of the optoelectronic polymers. Such an approach leads to the opportunity for a flexible and adjustable system which may solve the relatively low photovoltaic efficiencies that these polymers commonly exhibit [12,13]. As a result, a wide range of applications based on composite materials consisting of graphene oxide in polymer matrices have been reported in the literature. These systems have already been used as membranes [14,15], energy devices [16], supercapacitors [17], drug delivery [18] flexible and/or wearable electronics [19] etc.

The non-covalent attachment between the conjugated polymer and the rGO has significant advantages. The rGO is added to the polymer solution and the mixture is agitated through sonication. This approach leads to the stabilization through non-covalent interactions which do not induce intrinsic changes in the chemical structures, electronic or mechanical properties of the rGO. The π-π interactions between the rGO and the conjugated backbone render the hybrid materials capable of being easily processed due to the rGO solubility. The exquisite properties presented on the specific hybrid copolymers have paved the way for their extensive use in photovoltaic devices using facile methods [20,21,22,23].

Herein, our focus was the synthesis of high band gap conjugated polymers [24] based on indacenothiophene and indacenodithienothiophene [25] through the Stille aromatic coupling method. These monomers play the role of donors, in *donor-acceptor* units with varying acceptor units. The characterization through Gel Permeation Chromatography (GPC) indicated the molecular features of the copolymers such as number average molecular weight and dispersity (Đ). In addition, we report the synthesis of graphene oxide from graphite powder using a modified Staudenmaier method [2] and its further reduction to obtain the reduced graphene oxide [3]. X-ray Diffraction Analysis (XRD) was carried out to assess the characteristic properties before and after the oxygen incorporation between the graphene oxide sheets. The preparation of mixtures consisting of conjugated polymers and reduced graphene oxide is expected to have a significant role in the fine tuning of their energy gaps and possibly revolutionize various electronic applications such as organic solar cells, OLED displays etc. The materials were mixed in an appropriate dispersing medium (ortho-dichlorobenzene or o-DCB) and sonicated for 6 h prior to their characterization. Specifically, the hybrid dispersions were morphologically characterized using Scanning Electron Microscopy (SEM) and Energy Dispersive Spectroscopy (EDS). Their optoelectronic properties were evaluated with UV-Vis Spectroscopy and Cyclic Voltammetry (CV). Also, the thermal stability of the involved materials (GO, rGO, conjugated copolymers, hybrid materials) was verified through thermogravimetric analysis (TGA).

## 2. Materials and Methods

### 2.1. Materials

All monomers (thiophene-based, difluorobenzothiadiazole and diketopyrrolopyrrole) and solvents (toluene, methanol, acetone, hexane, acetonitrile, ortho-dichlorobenzene) were purchased from Sigma-Aldrich (St. Louis, MO, USA) and used without further purification. All reagents for the preparation of the samples [tris (dibenzylideneacetone)dipalladium (0) or Pd_2_dba_3_, tri (o-tolyl)phosphine or P (o-Tol)_3_, powdered graphite, sulfuric acid, nitric acid, potassium chlorate powder and sodium borohydride or NaBH_4_] were purchased from Fluka (Fluka Chemie GmbH, Buchs, Switzerland) and used without additional manipulations.

### 2.2. Methods

Number average molecular weights (${\bar{M}}_{n}$) and dispersity indices (Đ) were determined with Gel Permeation Chromatography (GPC) at 150 °C on a high temperature PL-GPC 220 system using a PL-GEL 10 μm guard column, three PL-GEL 10 μm Mixed-B columns and o-DCB as the eluent. The instrument (Agilent Technologies, St. Clara, CA, USA) was calibrated with narrow polystyrene standards (Mp ranged from 4830 g/mol to 3,242,000 g/mol).

UV/vis spectra were measured on a Cary 5000 UV-Vis-NIR (Agilent Technologies, St. Clara, CA, USA) in dual beam mode.

Raman spectroscopy studies were conducted through a micro-Raman RM 1000 Renishaw system (Renishaw, Wotton-under-Edge, UK). The power of the laser was 30 mW and a 2 μm focus spot was utilized.

X-ray diffraction (XRD) measurements were carried out in a Bruker D8 Advance (Bruker, Billerica, MA, USA) with Bragg–Brentano geometry with LYNXEYE detector in 2θ range from 2° to 30°. For the X-rays, a Cu-K_α_ wire was used, resulting in a radiation of 1.5406 Å wavelength.

Thermogravimetric analysis (TGA) was carried out in a Perkin Elmer Pyris-Diamond instrument (PerkinElmer, Inc., Waltham, MA, USA). Approximately 10 mg of each sample (graphene oxide, reduced graphene oxide, pristine conjugated copolymers and final hybrid materials) was heated from 40 °C to 800 °C following a step of 5 °C/min under nitrogen atmosphere.

Scanning Electron Microscope (SEM) images were obtained on gold coated samples (Polaron SC7620 sputter coater, Thermo VG Scientific, Waltham, MA, USA) using a JEOL JSM6510LV scanning electron microscope equipped with an INCA PentaFETx3 (Oxford Instruments, Abingdon, UK) energy dispersive X-ray (EDX) spectroscopy detector. Data acquisition was performed with an accelerating voltage of 20 kV and 60 s accumulation time, respectively. The samples were prepared using an appropriate solvent for solution casting and then dried overnight in a vacuum oven. Prior to the morphological characterization a gold/palladium target was used to sputter the samples utilizing the SC7620 Mini Sputter Coater/Glow Discharge System (Quorum technologies, Sacramento, USA). Also, additional experiments were conducted on a specific copolymer (copolymer-C2 with 5 wt% and 10 wt% rGO) in order to study its morphological behavior in thin film state. The samples were prepared using spin coating onto silicon wafer under ambient conditions (spinning velocity ~3000 rpm for approximately 30 s, polymer solution in o-DCB concentration equal to 3%).

Voltammetric Experiments were performed with a PGSTAT12 electrochemical analyzer (Metrohm Autolab, Utrecht, Netherlands) in a single compartment three-electrode cell. Bare or modified GCEs (IJ Cambria, Swansea, UK) were used as working electrodes and a platinum wire served as the auxiliary electrode. The reference electrode was an Ag/AgCl 3M KCl (IJ Cambria, Swansea, UK) electrode and all potentials hereafter are quoted to the potential of this electrode. Cyclic Voltammograms (CVs) were recorded in 0.1 mol L^−1^ Bu_4_NBF_4_ in solvent mixture acetonitrile (ACN):o-DCB at a scan rate of 0.05 V s^−1^. All potentials are quoted after the Fc/Fc^+^ redox couple as explained in the experimental section.

### 2.3. Synthesis of Hybrid Materials

Three (3) conjugated copolymers using thiophene-based and difluoro benzothiadiazole or diketopyrrolopyrrole as monomers were synthesized based on the Stille aromatic coupling in a process that has been already described by our group [26] in order to be used as matrices for the preparation of hybrid materials. In a three-neck round bottom flask, fitted with a condenser, 3 cycles of argon/vacuum were conducted. Afterwards, the donor- and the acceptor-type monomers were added along with Pd_2_dba_3,_ which is the catalyst (22.90 mg), and P (o-Tol)_3_ as a ligand (60.87 mg) and 3 cycles of argon/vacuum were carried out again. Finally, toluene (20 mL) was added, and 3 cycles of argon/vacuum were again performed. The reaction mixture is allowed to react for at least 48 h at 120 °C.

Upon completion of the polymerization, the synthesized material was precipitated in 500 mL of methanol and filtered on a paper filter. The filter was placed in a Soxhlet type device and solvent cleaning of different polarity starts. Initially, the polymer was purified with methanol (250 mL) to remove any catalyst residues, then with acetone (250 mL) to remove any unreacted monomers, followed by hexane (250 mL) to remove any oligomers and finally with chloroform (250 mL) to extract the desired copolymer. The chloroform fraction was concentrated on a rotary evaporator to a volume of approximately 50 mL and the copolymer was precipitated in 800 mL of methanol. The precipitated copolymer was collected by vacuum filtration and dried for 24 h on a PTFE filter. Further drying of the polymer in a vacuum oven at 40 °C for 9 h led to a yield of 0.86 g of the final copolymer. The synthetic procedure and chemical structures of the synthesized conjugated copolymers are presented in the Supporting Information for clarification reasons (Scheme S1). The molecular characteristics of the copolymers were specified using GPC and the results are summarized in Table S1 (Supporting Information). The three different molecular weight copolymers are abbreviated as C1, C2 and C3 respectively as evident in Table S1 in the supporting information. In addition, in Figure S1 the GPC curves with respect to the different copolymers are presented.

### 2.4. Synthesis of Graphene Oxide and Reduction to the Desired rGO

Graphene oxide was synthesized with the modified Staudenmaier method which is widely established [2]. In a typical synthesis, powdered graphite (5 g, purum powder ≤ 0.2 mm; Fluka) was added to a mixture of concentrated sulfuric acid (200 mL, 95–97 wt%) and nitric acid (100 mL, 65 wt%) while cooling in an ice-water bath. Potassium chlorate powder (100 g, purum > 98.0%; Fluka) was added to the mixture gradually while stirring and cooling. The reaction was quenched after 18 h by pouring the mixture into distilled water and the oxidation product was washed until the pH reached a value of 6.0 and finally dried at room temperature. The resulting GO was re-dispersed in 200 mL distilled water under agitation for 24 h. Then, 50 mL of an NaBH_4_ aqueous solution (8 mg/mL) were added and the dispersion was agitated for another 24 h [3]. Finally, the dispersion was centrifuged for five (5) times and the sediment was left to dry on a glass substrate. Using this procedure rGO was efficiently prepared.

### 2.5. Preparation of the Hybrid Dispersions

Many solvents have been proposed as appropriate dispersion mediums for rGO [27,28]. High-boiling point solvents showcase an outstanding performance in terms of stability. Our choice of dispersing rGO in o-DCB was two-fold: firstly to produce extremely stable dispersions of rGO and secondly it is a common solvent of conjugated polymers of the indaceno- type. Thus, we have prepared nine (9) different dispersions of hybrid materials using sonication for 6 h in each of the three different copolymers with 3, 5 and 10% wt rGO loading respectively as evident in Table 1.

## 3. Results and Discussion

### 3.1. Raman and X-ray Diffraction Analysis

Raman spectroscopy was performed to study the oxidation of pristine graphite to GO and its further reduction to rGO. In Figure S2 in the Supporting Information the Raman spectra of the two allotropic forms of graphene oxide obtained are illustrated. In the GO spectrum, the two major peaks D and G are 1310 cm^−1^ and 1590 cm^−1^ respectively possess quite large deviations and structural deficiencies in relation to graphene due to the existence of the oxygen groups that alter sp^2^ hybridization and therefore its structure. The imperfections are represented by the I_D_ / I_G_ ratio, which equals to 1.99, a value that is justified by the oxidation process of graphite. Correspondingly for the case of rGO, D and G peaks at 1301 cm^−1^ and 1575 cm^−1^ respectively show the structural defects from the non-selective removal of the oxygen groups due to the reduction which induces gaps along the structure. In this case, the I_D_/I_G_ ratio increases to 2.23, indicating the successful rGO reduction [29].

Concerning the XRD measurements conducted on GO and rGO a clearcut difference on the first reflection is evident in the XRD spectra presented in Figure S3. Taking into consideration the primary reflection (001) and by applying Bragg’s relationship one concludes that the reduction of the original oxygen groups leads to a change in the arrangement of the graphene sheets. Specifically, d_(001)_ GO equals to 7.4 Å while d_(001)_ rGO to 9.1 Å.

The successful chemical modification is verified by the increase of the neighboring graphite sheets distance as indicated by the synergy of literature [30] and experimental results. In Figure S3 the comparative diffraction diagram for GO and rGO is presented showcasing a distinct difference on the first reflection which reveals the larger interstitial space between the rGO sheets. The specific properties are desirable for the preparation of nanocomposite materials due to the capability of the conjugated polymer to be accommodated between the interstitial spaces of the laminar material.

TGA experiments were performed to study the thermal behavior of both the GO and the rGO. A minimal weight loss (8%) at approximately 100 °C is evident and corresponds to the water molecules, while functional groups (-O(CO)-, -OH, -O-) are decomposed at approximately 230 °C, leading to a second significant weight loss (30%). The graphitic lattice decomposition (50% weight loss) took place at approximately 500 °C (Figure S4A in the Supporting Information). The TGA studies on the rGO revealed a weight loss at approximately 10% due to the removal of moisture. The remaining functional groups (-O(CO)-, -OH, -O-) after the chemical modification are removed at approximately 190 °C leading to 20% weight loss which is less than the one observed in the GO further verifying the successful chemical modification. The graphitic lattice decomposition occurred at approximately 530 °C indicating higher thermal stability of the rGO due to the chemical modification of the functional groups (Figure S4B in the Supporting Information). The thermograph of the pristine conjugated copolymer (C3) and hybrid materials consisting of the conjugated copolymer C3 with different weight percentages of rGO are also presented in Figure S4C–F. The original decomposition of the pure conjugated copolymer took place at approximately 238 °C (Figure S4C). It is evident that the decomposition of the conjugated copolymer with 3 wt% of rGO initiated at 242 °C (Figure S4D), while for the 5 wt% rGO at 250 °C (Figure S4E) and for the 10% rGO at 260 °C (Figure S4F) indicating that the higher the rGO ratio the greater the thermal stability of the final hybrid material. Coherent behavior was also observed in the remaining samples. Note that, the materials with the highest percentage of rGO not only showcased the highest stability but also less weight loss which is in accordance to previous studies [31].

### 3.2. Morphological Characterization

**Figure 1:**
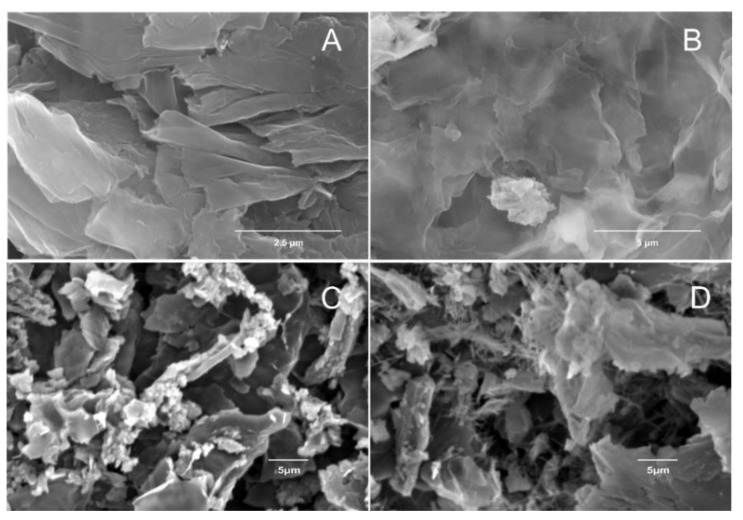
SEM images of the GO and rGO in film (**A**,**B**) and in solid (**C**,**D**) forms.

The morphological characterization of GO, rGO and the final hybrid materials was performed through SEM measurements. In Figure 1 the difference in the nanosheets of GO (Figure 1A) and rGO (Figure 1B) in the dispersion form (an aqueous dispersion for GO and an o-DCB dispersion for rGO which was left to dry at an elevated temperature overnight under vacuum) is depicted. The SEM micrographs presented in Figure 1C,D correspond to the solid form of the GO and the rGO respectively, indicating an apparent introduction of abnormalities in the rGO morphology compared to the defect-free GO, which are attributed to the reduction process as vacancies that occur in the lattice during the removal of the oxygen groups. From the SEM images it is evident that the conversion of the graphite morphology to graphene oxide was accomplished successfully maintaining its lamellar formation. Also, it is straightforward that the film and solid forms exhibit significant alternations due to the re-dispersion of GO and rGO in o-DCB for the case of film state.

**Figure 2:**
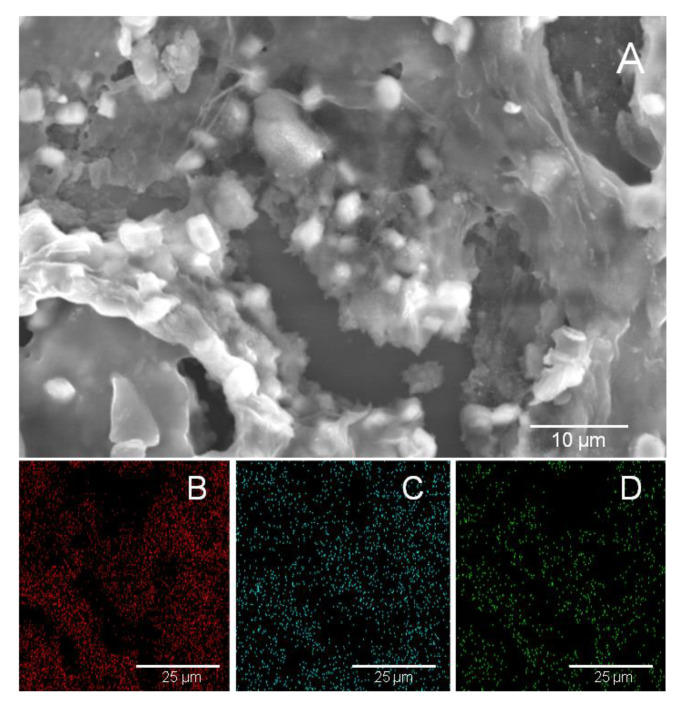
SEM images of a hybrid system with the Copolymer 2 and the rGO (**A**). There is an apparent intercalation that is supported with the aid of EDS, where the elemental mapping analysis confirms the existence of Carbon (**B**), Sulfur (**C**) and Oxygen (**D**).

In Figure 2 the SEM micrograph in conjunction with the elemental mapping analysis of the hybrid material which is constituted by copolymer 2 (C2) with 5 wt% rGO is demonstrated. The interaction of the conjugated copolymer with the rGO due to π-π stacking led to the intercalation of the copolymer between the graphene nanosheets, a behavior that has been reported for similar layered systems [32] (Figure 2A). This is further supported by the EDX in which the elemental mapping analysis confirmed the existence of carbon (Figure 2B) (a common element of the hybrid system), sulfur (Figure 2C) which is derived from the thiophene groups in the copolymer structural unit and oxygen (Figure 2D), which is solely found in rGO and verifies the successful preparation of the hybrid material. The SEM/EDX data obtained from the different copolymers using 3, 5 and 10 wt% of rGO, showcased similar results, suggesting the non-covalent attachment of the rGO on the conjugated copolymers even in low concentration values.

Interestingly enough, organic cells-related applications are based on polymer films and therefore the morphological characterization of the hybrid material which is comprised by the C2 copolymer with either 5 wt% or 10 wt% rGO were conducted in thin film state. The spin coated samples were approximately ~100 nm thick and the SEM studies indicated similar structures to the ones obtained during bulk characterization. The results are promising for photovoltaic applications, but more comprehensive studies are required. Two representative SEM micrographs are presented in the Supporting Information (Figure S5a,b).

### 3.3. Optoelectronic Properties

**Figure 3:**
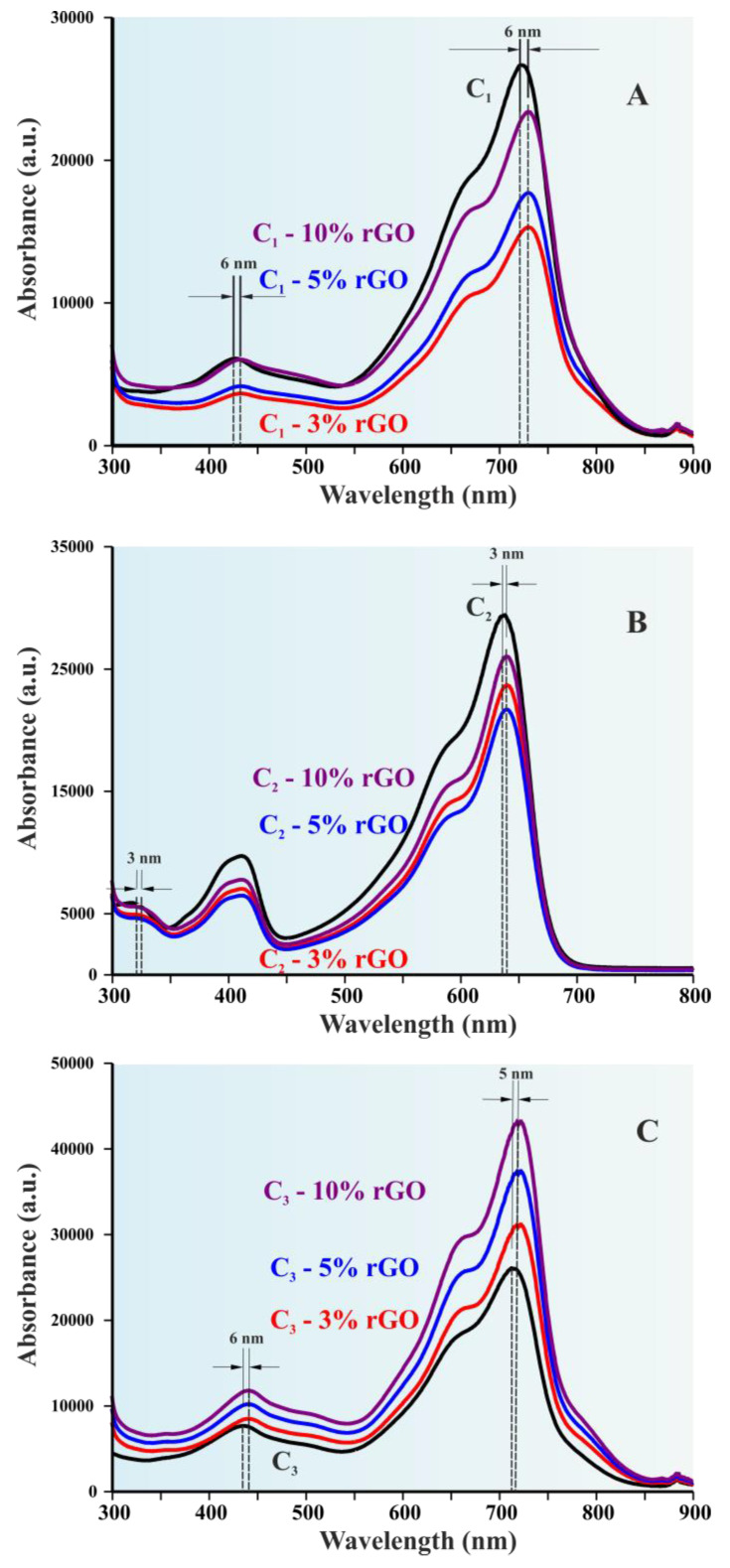
UV-Vis spectra of the: (**A**) C1 systems; (**B**) C2 systems; and (**C**) C3 systems. Distinct wavelength shifts can be observed in every system for even a minimum rGO addition, at (red line) C-3% rGO, (blue line) C-5% rGO, and (purple line) C-10% rGO.

In Figure 3 the UV-Vis spectra obtained from 300–900 nm for the three pristine copolymer systems and the hybrid materials with different rGO concentration are given. For copolymer 1 (C1) (Figure 3A) two distinct peaks at 423 nm and 723 nm can be clearly identified. With the addition of rGO, the first absorption peak was shifted from 423 nm to 429 nm while the second peak from 723 nm to 729 nm. This phenomenon indicates that even a relatively small (3% wt) addition of rGO is able to alter the absorption maxima of the copolymer and hence its energy gap. This phenomenon is also observed in samples with higher rGO concentration, which also exhibit similar displacements at a higher absorption intensity. Consistently, similar results were recorded for copolymer C2 (Figure 3B) which exhibits three absorption maxima (637 nm, 411 nm and 319 nm) but the shift only occurs for two maxima, since the peak at 637 nm shifts to 640 nm and the peak at 319 nm shifts to 322 nm. Finally, copolymer C3 (Figure 3C) demonstrates a similar shift in wavelength as C1 (from 713 nm to 719 nm and from 434 nm to 440 nm), thus reaffirming the impact of the rGO addition on the optical properties.

Cyclic voltammetry was performed to correlate the oxidation and the reduction potential onsets with the respective HOMO (Highest Occupied Molecular Orbital) and LUMO (Lowest Unoccupied Molecular Orbital) levels as it has been previously described for such conjugated copolymers by our group [33]. The cyclic voltammograph of ferrocene in acetonitrile (ACN) was used as reference for the copolymers and is presented in the Supporting Information (Figure S6). The oxidation and reduction potentials were recalibrated versus the Fc/Fc^+^ redox couple and the corresponding HOMO and LUMO levels were calculated using the following equations:

E_HOMO_ = 5.1 + E^ox^_onset_, (1)

E_LUMO_ = 5.1 + E^red^_onset_ (2)

**Figure 4:**
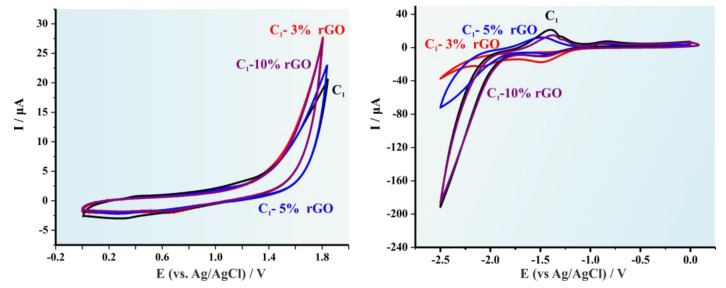
Cyclic voltammograms of the C1 systems for both the oxidation and the reduction process. in 0.1 mol L^−1^ Bu_4_NBF_4_. Scan rate 0.050 V s^−1^. Signals presented as: (red line) C1-3% rGO, (blue line) C1-5% rGO, and (purple line) C1-10% rGO.

**Figure 5:**
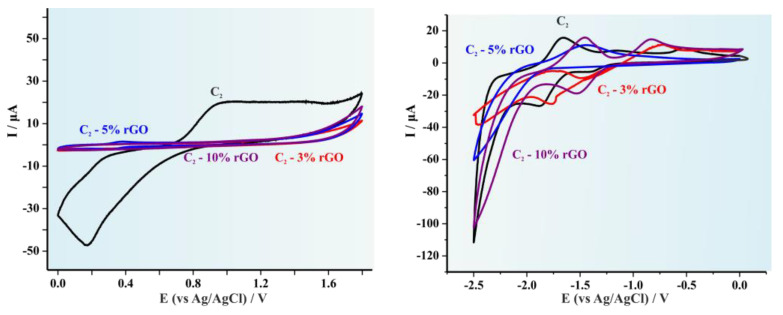
Cyclic voltammograms of the C2 systems for both the oxidation and the reduction process. in 0.1 mol L^−1^ Bu_4_NBF_4_. Scan rate 0.050 V s^−1^. Signals presented as: (red line) C3-3% rGO, (blue line) C3-5% rGO, and (purple line) C3-10% rGO.

**Figure 6:**
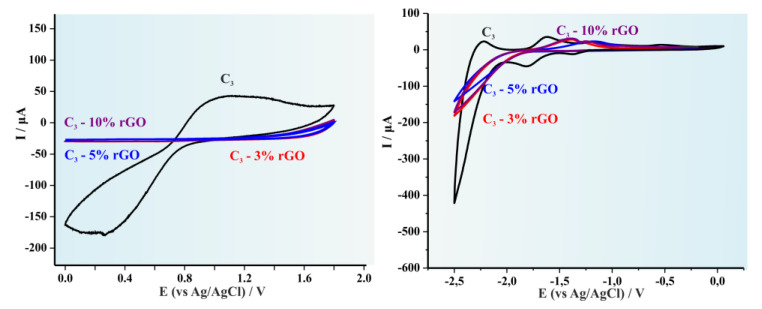
Cyclic voltammograms of the C3 systems for both the oxidation and the reduction process. in 0.1 mol L^−1^ Bu_4_NBF_4_. Scan rate 0.050 V s^−1^.

The cyclic voltammographs (CVs) for the pristine C1 copolymer and the produced hybrid materials after the addition of 3, 5 and 10% wt rGO respectively are illustrated in Figure 4. It is apparent that for lower rGO concentrations (3 and 5 wt%) a significant alteration on the redox potentials is experienced and therefore to the HOMO and LUMO levels, while in higher concentration (10 wt%) the redox potentials are similar to those obtained for the pristine copolymer [34]. This effect is observed for all three hybrid systems since it is also evident in Figure 5 (C2) and in Figure 6 (C3). Table 1 summarizes the results provided from the CVs as well as the calculated HOMO, LUMO levels and the electrochemical band gap (E_g_).

## 4. Conclusions

Graphene oxide (GO) and reduced graphene oxide (rGO) were synthesized and successfully combined with indacenodithiophene- and indacenodithienothiophene-based conjugated copolymers for the preparation of hybrid materials in order to study the alteration on their optoelectronic properties due to non-covalent binding (π-π stacking) with rGO. The conjugated copolymers were synthesized through Stille aromatic coupling. Staudenmaier reaction was utilized for the synthesis of GO, which was further reduced for the preparation of rGO. The conjugated polymers were mixed using different ratios of rGO and were ultrasonicated in ortho-dichlorobenzene that constitutes an excellent dispersion medium for each system, to produce stable dispersions. To identify the successful synthesis of GO and rGO, morphological and structural characterization was performed using Scanning Electron Microscopy (SEM), Raman spectroscopy and X-Ray Diffraction analysis. UV-Vis absorption spectrophotometry was used to determine the optical properties of the pristine copolymers and the hybrid materials. The experiments indicated a significant wavelength shift in the absorption maxima for all rGOs of the order of 3–6 nm, which justifies the alteration and fine-tuning of the optical properties of the copolymers with the aid of rGO. Finally, electrochemical characterization was performed via Cyclic Voltammetry (CV) to determine the energy levels of copolymers and mixtures. From the determination of oxidation and reduction potentials and the calculated HOMO and LUMO levels it is concluded that small percentages of rGO (3–5 wt%) can alter the energy levels of the copolymers and ultimately decrease the electrochemical Energy gap (E_g_), while larger concentrations of rGO can return these attributes to their original state. This suggests that the specific hybrid materials are quite promising for applications such as organic solar cells, where a tunable band gap and wavelength are of strategic importance.

## Figures and Tables

**Table 1:** The oxidation and reduction potentials from the respective cyclic voltammograms for each system (recalibrated versus Fc/Fc^+^) and the corresponding HOMO, LUMO and band gaps.

Copolymer	${\bar{M}}_{n}$ (kDa) *	E^ox^_onset_ (V vs. Fc/Fc+)	E^red^_onset_ (V vs. Fc/Fc+)	E_HOMO_ (eV)	E_LUMO_ (eV)	Eg^ec^
1	128	0.05	−2.01	−5.15	−3.09	2.06
3 %rGO		−0.18	−1.73	−4.92	−3.37	1.55
5 %rGO		−0.13	−2.09	−4.88	−3.01	1.87
10 %rGO		0.11	−1.95	−5.21	−3.15	2.06
2	153	0.13	−2.35	−5.23	−2.75	2.48
3 %rGO		−0.16	−1.88	−4.94	−3.22	1.72
5 %rGO		−0.14	−1.79	−4.96	−3.31	1.65
10 %rGO		0.44	−2.07	−5.54	−3.03	2.51
3	150	0.55	−2.04	−5.65	−3.06	2.59
3 %rGO		0.03	−1.94	−5.13	−3.16	1.97
5 %rGO		0.01	−1.59	−5.11	−3.51	1.60
10 %rGO		0.17	−1.97	−5.27	−3.13	2.14

* Number average molecular weights were calculated from Gel Permeation Chromatography measurements. (For additional information, refer to Supporting Information).

## Data Availability

The data presented in this study are available upon request from the corresponding author.

## Supplementary Materials

The following supporting information can be downloaded at: https://www.mdpi.com/article/10.3390/polym14235292/s1, Scheme S1: Chemical reactions and structures of: (**A**) indacenothiophene-alt -3,6-bis(5-bromothiophen-2-yl)-2,5-bis(2-octyldodecyl)pyrrolo [3,4-c]pyrrole (IDT and Dibromo-DPP) or C1; (**B**) indacenothiophene -alt-4,7-dibromo-5,6-difluorobenzo[1,2,5]thiadiazole (IDT and difluoro-BTD) or C2; and (**C**) Indacenodithienothiophene-alt-3,6-bis(5-bromothieno[3,2-b]thiophen-2-yl)-2,5 bis(2octyldodecyl)pyrrolo[3,4-c]pyrrole (IDTT and Dibromo-DPP) or C3 copolymers; Figure S1: Gel Permeation Chromatography (GPC) curves of the three different copolymers where the black line corresponds to the C1 copolymer, the red line to the C2 copolymer and the blue line to the C3; Table S1: Molecular characteristics of the three different copolymers as directly calculated from gel permission chromatography; Figure S2: Raman Spectra obtained for GO and rGO. Both spectra represent materials with a great number of defects which is evident by the respective I_G_/I_D_ ratio. The red curve corresponds to the rGO while the black to the GO; Figure S3: XRD measurements for GO and rGO. The red curve corresponds to the rGO while the black to the GO; Figure S4: TGA thermograms under nitrogen atmosphere corresponding to: (**A**) GO; (**B**) rGO; (**C**) C3 copolymer; (**D**) C3 copolymer with 3 wt% rGO; (**E**) C3 copolymer with 5 wt% rGO and F) C3 copolymer with 10 wt% rGO; Figure S5: SEM images corresponding to the C2 copolymer with (**A**) 5 wt% rGO; and (**B**) 10 wt% rGO. The samples were prepared using the spin coating technique leading to thicknesses of approximately 100 nm; Figure S6: Cyclic voltammogram of ferrocene in ACN which was used as reference for the copolymers
