# Supplementary material for: Synthesis and Characterization of Hybrid Materials Derived from Conjugated Copolymers and Reduced Graphene Oxide
Source: Polymers (Basel). 2022 Dec 3;14(23):5292. doi: 10.3390/polym14235292 (PMC9737337; doi:10.3390/polym14235292)
Supplement: Supplementary file 1 [file polymers-14-05292-s001.zip › polymers-2052027-supplementary.pdf]

Supporting Information

Synthesis and Characterization of Hybrid Materials Derived from Conjugated Copolymers and Reduced Graphene Oxide

Alexandros Ch. Lazanas[1], Athanasios Katsouras[1], Michael Spanos[1, 2], Gkreti-Maria Manesi[1], Ioannis Moutsios[1,3], Dmitry V. Vashurkin[4,5], Dimitrios Moschovas[1], Christina Gioti[1], Michail A. Karakassides[1], Vasilis G. Gregoriou[2], Dimitri A. Ivanov[3,4,5], Christos L. Chochos[1, 2*], and Apostolos Avgeropoulos[1,4,5*]

[1] Department of Materials Science Engineering, University of Ioannina, University Campus, Ioannina 45110, Greece
[2] National Hellenic Research Foundation (NHRF), 48 Vassileos Constantinou Avenue, Athens 11635, Greece
[3] Institut de Sciences des Matériaux de Mulhouse—IS2M, CNRS UMR7361, 15 Jean Starcky, 68057 Mulhouse, France
[4] Faculty of Chemistry, Lomonosov Moscow State University 9MSU), GSP-1, 1-3 Leninskiye Gory, 119991 Moscow, Russia
[5] Institute of Problems of Chemical Physics, Russian Academy of Sciences, Chernogolovka, 142432 Moscow, Russia
* Correspondence: chochos@eie.gr; aavger@uoi.gr; Tel.: +30-26-5100-9001

A

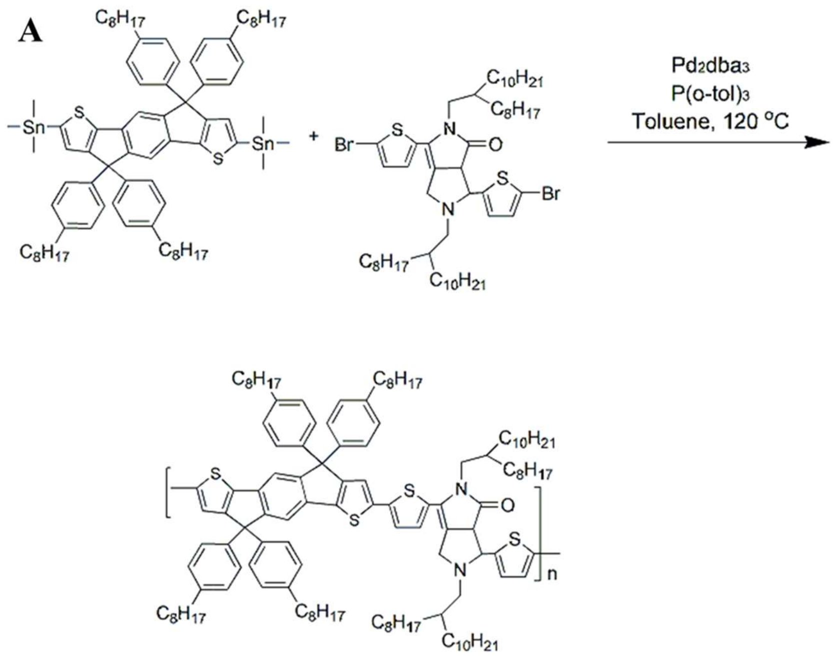

B

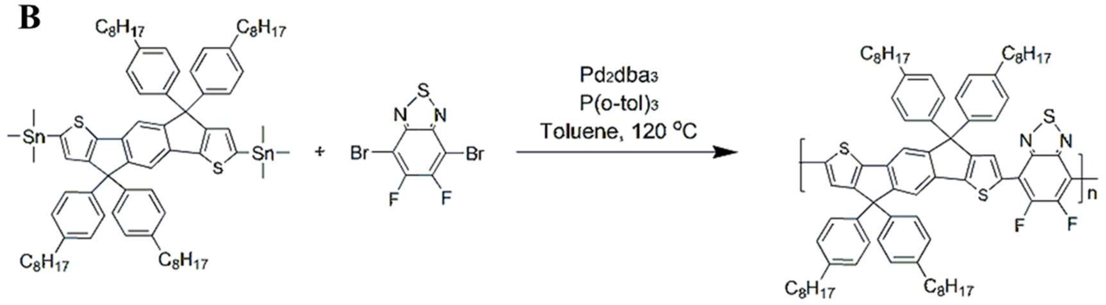

C

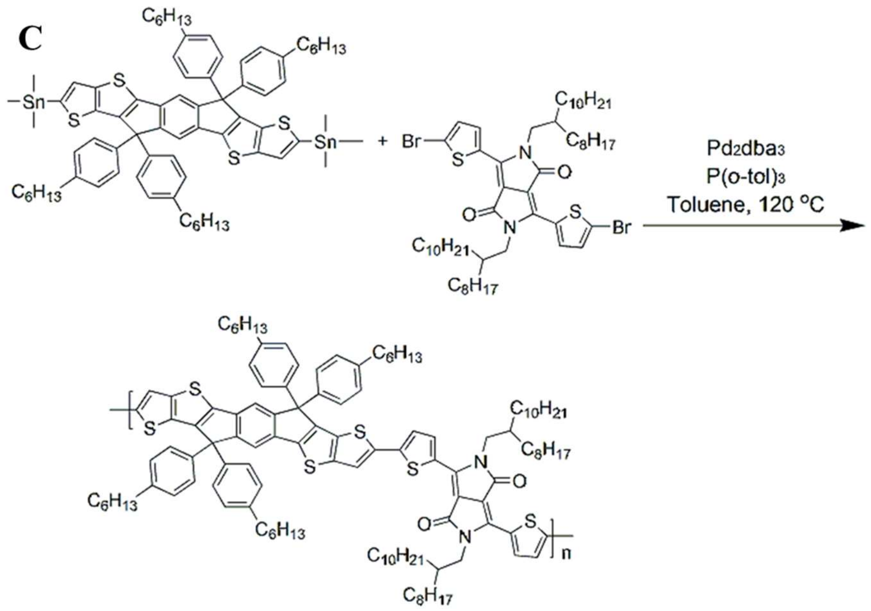

Scheme S1. Chemical reactions and structures of: A) indacenothiophene -alt -3,6-bis(5-bromothiophen-2-yl)-2,5-bis(2-octyldodecyl)pyrrolo[3,4-c]pyrrole (IDT and Dibromo-DPP) or C1, B) indacenothiophene -alt-4,7-dibromo-5,6-difluorobenzo[1,2,5]thiadiazole (IDT and difluoro-BTD) or C2 and C) Indacenodithienothiophene-alt-3,6-bis(5-bromothieno[3,2-b]thiophen-2-yl)-2,5 bis(2octyldodecyl)pyrrolo[3,4-c]pyrrole (IDTT and Dibromo-DPP) or C3 copolymers.

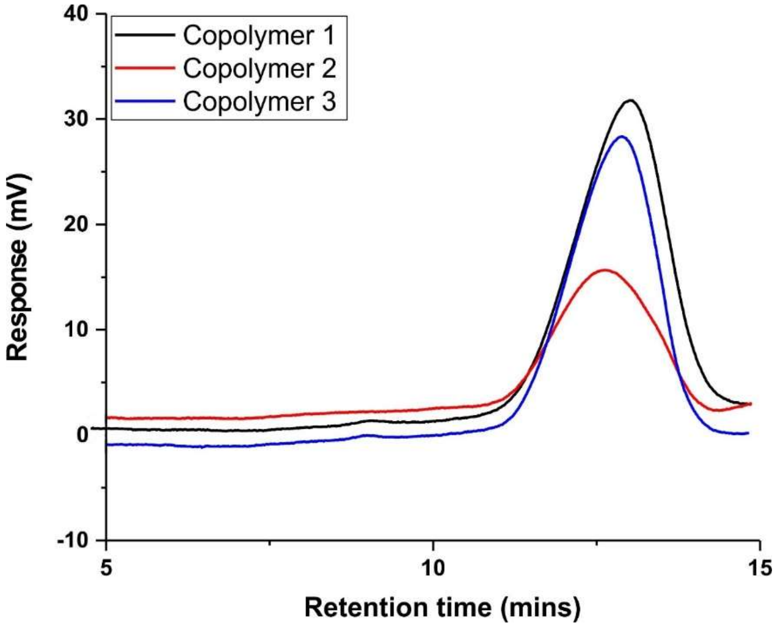

Figure S1. Gel Permeation Chromatography (GPC) curves of the three different copolymers where the black line corresponds to the C1 copolymer, the red line to the C2 copolymer and the blue line to the C3.

Table S1. Molecular characteristics of the three different copolymers as directly calculated from gel permission chromatography.

Copolymer	Average Molecular Weight per Number kDa	Average Molecular Weight per Weight kDa	Dispersity Index (Đ)
**C1**	128	302	2.36
**C2**	153	379	2.48
**C3**	150	328	2.19

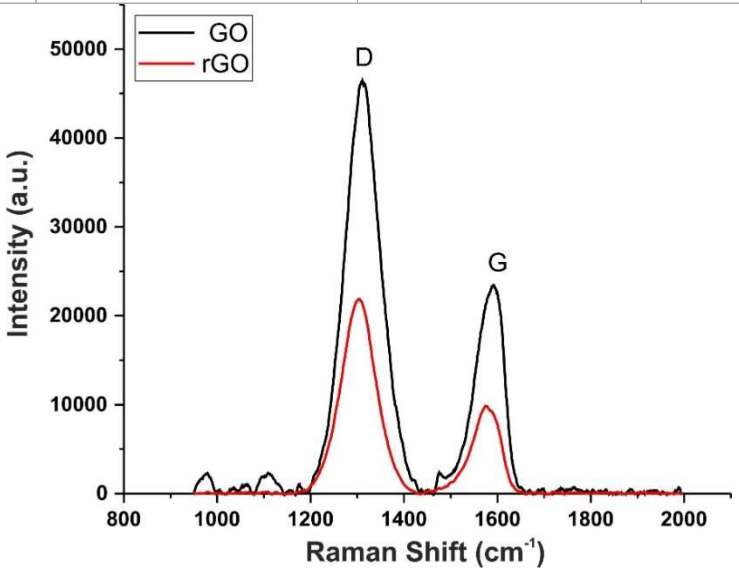

Figure S2. Raman Spectra obtained for GO and rGO. Both spectra represent materials with a great number of defects which is evident by the respective I_G / I_D ratio. The red curve corresponds to the rGO while the black to the GO.

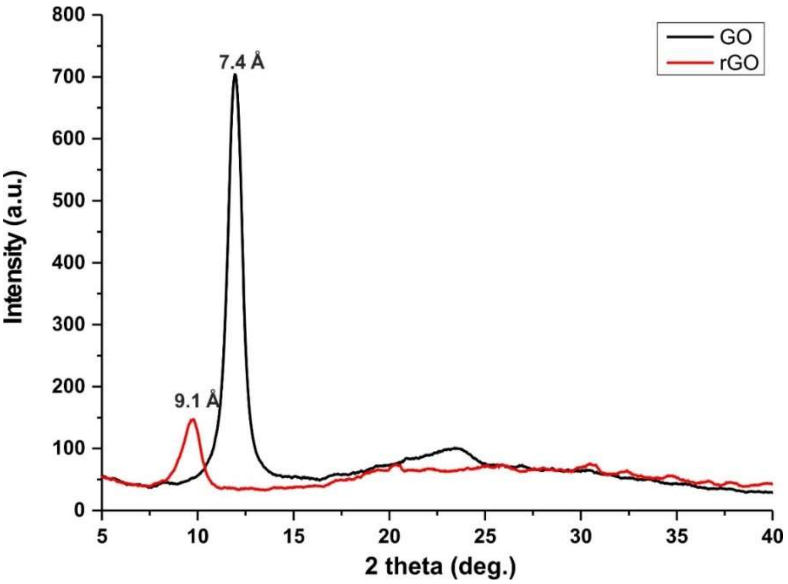

Figure S3. XRD measurements for GO and rGO. The red curve corresponds to the rGO while the black to the GO.

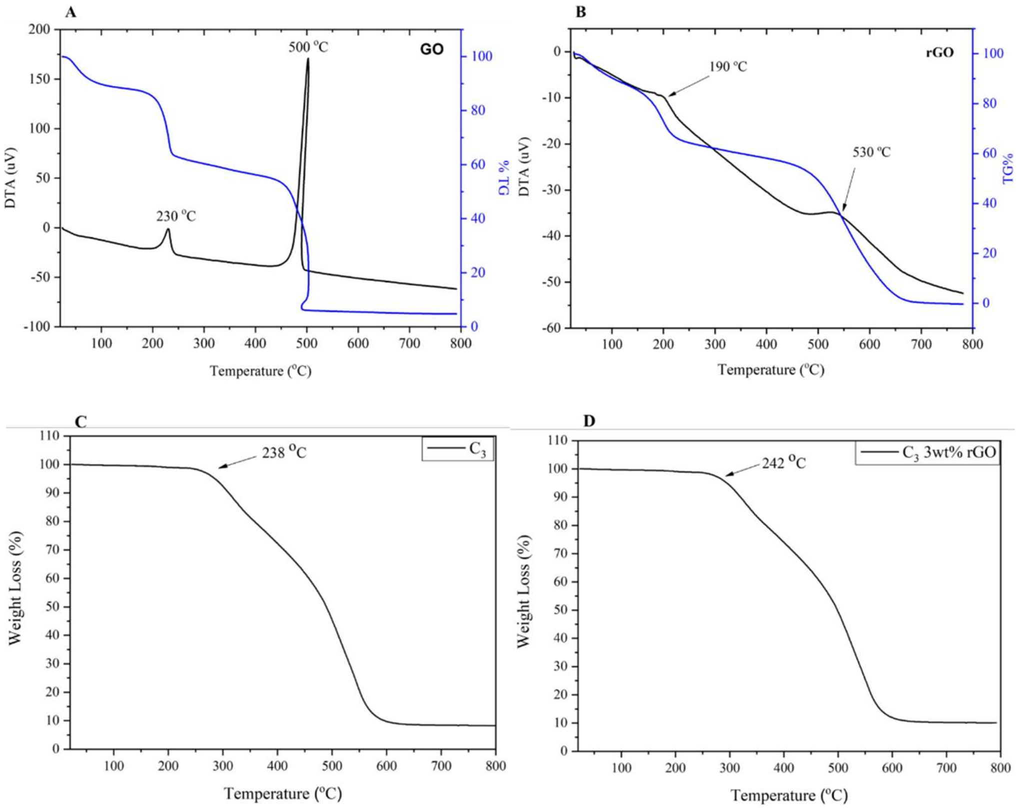

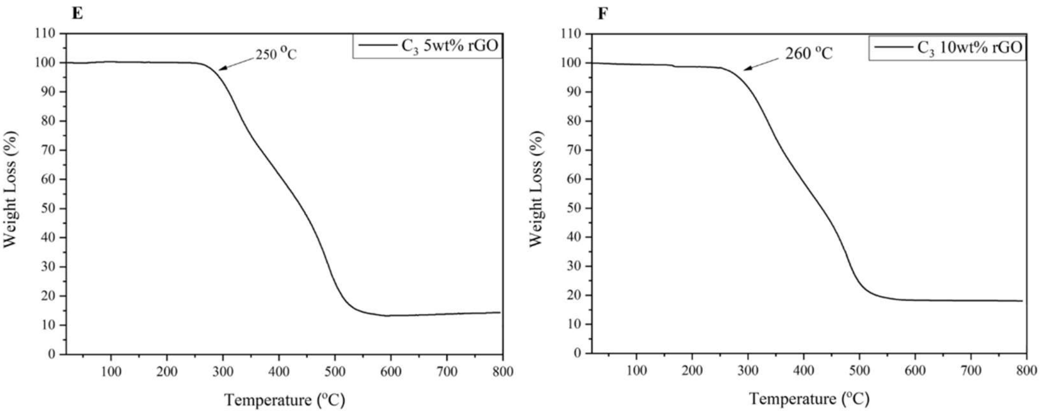

Figure S4. TGA thermograms under nitrogen atmosphere corresponding to: A) GO, B) rGO, C) C3 copolymer, D) C3 copolymer with 3 wt% rGO, E) C3 copolymer with 5 wt% rGO and F) C3 copolymer with 10 wt% rGO.

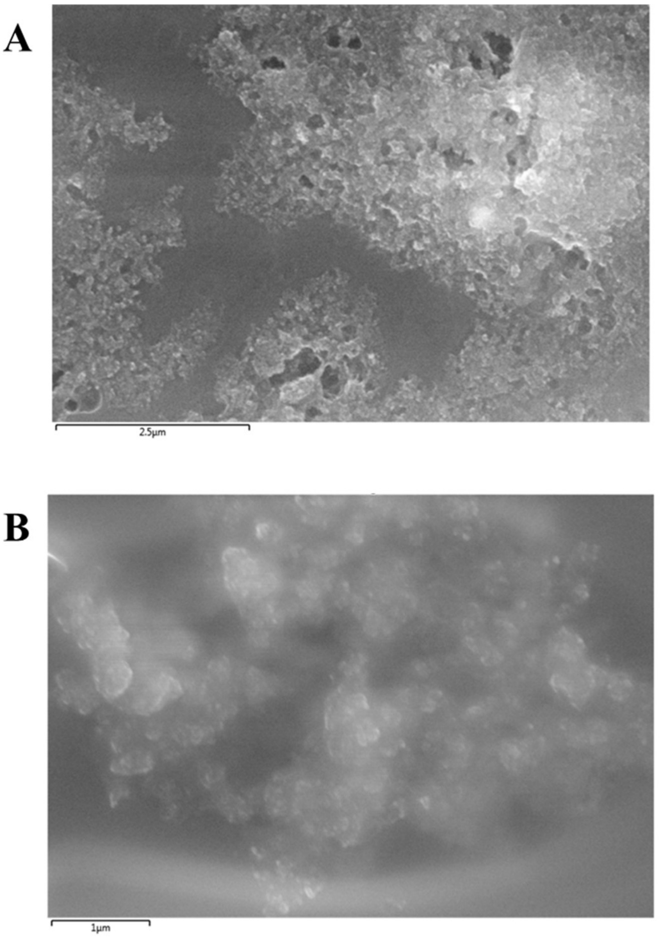

Figure S5. SEM images corresponding to the C2 copolymer with A) 5 wt% rGO and B) 10 wt% rGO. The samples were prepared using the spin coating technique leading to thicknesses of approximately 100 nm.

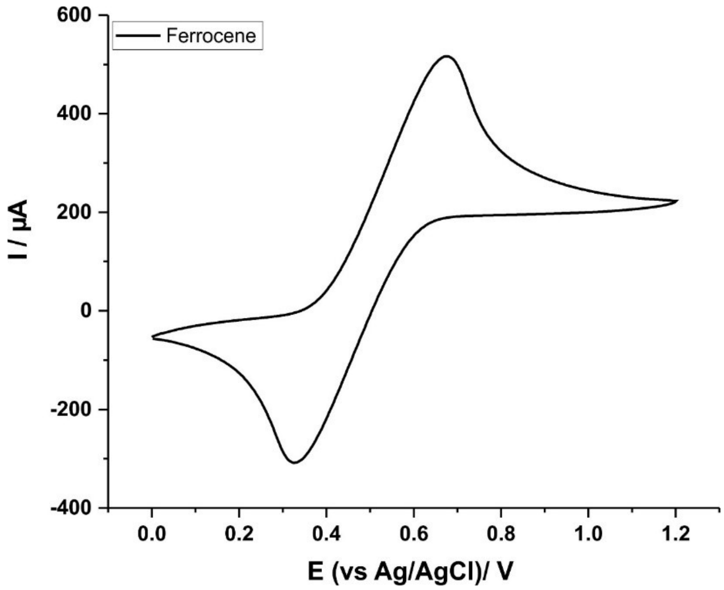

Figure S6. Cyclic voltammogram of ferrocene in ACN which was used as reference for the copolymers.
